# Editor’s Book Table

**Published:** 1869-10

**Authors:** 


### Editor's Book Table.

[NOTE.—All works reviewed in the columns of THE CHICAGO MEDICAL JOURNAL may be found in the extensive stock of W. B. Keen & Cooke, whose catalogue of medical books will be seen in the present number.]

*A Treatise on the Diseases and Surgery of the Mouth, Jaws, and Associate Parts.* By JAMES E. GARRETSON, M.D., D.D.S., late lecturer on Anatomy and Surgery in the Philadelphia School of Anatomy; late Prof. of the Principles and Practice of Surgery in the Philadelphia Dental College, etc., etc. Illustrated with steel plates and numerous wood cuts. Philadelphia: J. B. Lippincott & Co., 1869. Pp. 700. $7.50. Chicago: W. B. Keen & Cooke.

A GLANCE at the headings of the forty-two chapters of this work will abundantly convince the reader of the great importance of the several subjects treated, and reading of the text will demonstrate that the author has accomplished the task proposed with consummate ability and fidelity. No dental surgeon can consider his library complete, or himself fully "posted," without this invaluable book. It is the most scholarly and at the same time practical exposition of the general subjects treated upon, that it has been our fortune in a long time to meet, even if it has ever been equaled. But it is by no means to be restricted in use and value to our *confreres*, the dentists. Aside from its discussion of the pathology and therapeutics of the teeth, about two-thirds of the space is devoted to elucidation of disorders topographically associated, and here the most accomplished surgeon will find much to interest and instruct. We are especially pleased with the multitudinous new and beautiful illustrations afforded. We do not hesitate to say that in this respect the book equals, if it does not surpass, any professional book up to this time issued from the American press. It affords us unusual pleasure to advise each of our readers to buy, read and rejoice over this really *new* book. We congratulate the author upon its undoubted immediate popularity and success.

*A Course of Practical Chemistry*, Arranged for the Use of Medical Students. By WILLIAM ODLING, M.B., F.R.S., Lecturer on Chemistry at St. Bartholomew's Hospital, etc. With Illustrations. From the fourth and revised London edition. Philadelphia: Henry C. Lea, 1869. Pp. 261. $2. Chicago: W. B. Keen & Cooke.

A CONVENIENT hand-book for the student, and for reference by the practitioner. It is well illustrated. In the present edition the new system of atomic weights and formulæ is employed, but no general changes in the nomenclature have been introduced.

*Electricity in its Relations to Practical Medicine.* By Dr. MORITZ MEYER, Royal Counselor of Health, etc. Translated from the third German edition, with notes and additions, by WILLIAM A. HAMMOND, M.D., late Surgeon-General U. S. A., Professor of Diseases of the Mind and Nervous System, and of Clinical Medicine in Bellevue Hospital, Medical College, etc., etc. New York: D. Appleton & Company, 1869. Pp. 497. $4.50. Chicago: W. B. Keen & Cooke.

THE fact that Prof. Hammond has deemed this treatise sufficiently meritorious to induce him personally to translate and annotate it, is a good indication of its intrinsic value. He states emphatically, in his prefatory note, that it is the best which has yet appeared on the subject. The profession is certainly to be congratulated on the acquisition of further light on matters which have heretofore been largely exiled to the domains of professsed quackery.

*A Text-Book of Practical Medicine, with particular reference to Physiology and Pathological Anatomy.* By Dr. FELIX VON NIEMEYER, Professor of Pathology and Therapeutics, Director of the Medical Clinic of the University of Tübingen. Translated from the seventh German edition, by special permission of the author, by George H. Humphreys, M.D., etc., and Charles E. Hackley, M.D., etc., (New York City.) New York: D. Appleton & Company, 1869. In two vols. Pp. 731 and 77ɔ. Cloth, $9. Sheep, $15.00. Chicago: W. B. Keen & Cooke.

THE fact that *seven* editions of Prof. Niemeyer's book were called for within ten years, and it had received the further indorsement of translation into most of the principal languages of modern Europe, encouraged the American translators in the belief that it would prove acceptable to readers in this country. The last German edition had been thoroughly revised and enriched by copious additions. Although numerous text-books upon practice exist in the English language, yet, as for many of the most important researches and discoveries, both in pathology and therapeutics, we are indebted to Germany, and the concise and well-digested epitome of the results of ten years of carefully recorded clinical observations by the most illustrious medical authorities of Europe, together with many valuable and practical deductions regarding the cause of disease, and application of remedies, presented in these volumes, can scarcely elsewhere be found assembled in any single work; therefore we agree with the worthy translators in the fitness of the task which they have faithfully accomplished.

Prof. Niemeyer boldly announces himself as a defender of empirical therapeusis. Although himself a brilliant and profound pathologist, he declares "that even the dazzling progress which pathology has made has been but of little use in therapeutics; that in spite of new discoveries, our present success at the bedside is scarcely more favorable than that of fifty years ago."

Nevertheless, it is easy to see that, notwithstanding this somewhat gloomy and discouraging view of affairs, the author is far more of an optimist than he is willing to admit, and his treatise proves the fact. It takes time for even the keenest observers, and the sturdiest students, to arrive at this foundation fact, that medical science is thus far perfecting mainly by exclusion of the false and inane rather than by accretion of the new. We are learning not so much a new therapeusis as excision from the old. "Truth advances only over ruins, yet unceasingly advances."

As an evidence of the proposition that the author's real sentiments are not expressed in the *pessimist* remark first quoted, we adduce this better view:

"My conviction is, that from the present state of knowledge, from our deeper insight into the origin and relation of symptoms from the improved accessories, by means of which we are now enabled to follow the various phases and modifications of disease, the prospect of obtaining sure and authentic therapeutic facts, by dint of accurate comparison of results, is not only by no means unfavorable, but, judging from present experience, is positively certain."

We do not find in this treatise any traces of the bold empiricism we might apprehend from some parts of the preface. But the strength of the book lies in its pathology, and its clear association of existent symptoms with existent lesions. There is a cheering absence of *twaddle* about "functional" diseases. We prize the work as a whole, and cordially commend its careful perusal.

*The Mechanism of Dislocation and Fracture of the Hip, with the Reduction of the Dislocations by the Flexion Method.* By HENRY J. BIGELOW, M.D., Professor of Surgery and Clinical Surgery in the Medical School of Harvard University; Surgeon of the Massachusetts General Hospital, etc., etc. With Illustrations. Philadelphia: Henry C. Lea, 1869. Pp. 150. $2.50. Chicago: W. B. Keen & Cooke.

THIS is a luminous exposition of the subject named in the title, put before the profession, by the publisher, in elegant style, both of paper and typography, with excellent illustrations. No writer could ask for a better dress in which to make a public appearance.

As suggested by the title, this is a scientific account of the successful treatment of dislocations and fractures of the hip, first, so far as we are aware, empirically practiced by the late venerable Nathan Smith; certainly as far back as 1815, and, possibly, earlier still, and more recently recalled to professional attention by Dr. Reid, of Rochester, New York. The father of the present writer was a private student of Prof. Smith during his connection with Dartmouth College, and the writer was instructed by him, in Prof. Smith's empirical method, at least eight or ten years before Dr. Reid's paper was read before the State Society of New York. So much for priority in the method.

To those who only wish to familiarize themselves with the principles and the method, we can refer Prof. Bigelow's essay as complete and entirely satisfactory. But in our sphere as journalists we are compelled to complain of the author as unfair to an unwarrantable extent in passing over, with slight notice, an essay which in every particular anticipates the explanations he affords. We allude to an essay which can not have escaped his notice, because on page 14 of his book he expressly refers to it. Although it has not escaped his notice that PROFESSOR GUNN, in 1853, published in pamphlet form a full account of the mechanism involved, it would appear that he did not read it very carefully, or he would not have stated, as he does on his page 14, that "Prof. Gunn maintains, in a paper upon this subject, that any untorn or 'undissected' portion of the capsular ligament is capable of producing the signs of hip and shoulder luxation." The fact is that Prof. Gunn expressly specifies the *anterior* and *inferior* portion of the capsular ligament, that part which Prof. Bigelow dignifies by the new name, the **Y** ligament. So far as we are able to see, this author has added nothing to Prof. Gunn's exposition of the subject, save only this new name of that portion of the capsular ligament — the *anterior* and *inferior* — heretofore known as the *ilio-femoral*, but which he christens as the **Y** ligament. In the subsequent editions, to which this little work, from its intrinsic merits, will assuredly pass, we trust Prof. Bigelow will do himself the honor, and Prof. Gunn the justice, to set this matter right before his readers.

*The Science and Art of Surgery.* Being a Treatise on Surgical Injuries, Diseases and Operations. By JOHN ERICHSEN, Senior Surgeon to University College Hospital, etc., etc. From the fifth enlarged and carefully revised London edition. Illustrated with six hundred and thirty engravings on wood. With additions by JOHN ASHHURST, Jr., A.M., M.D., Vice-President of the Philadelphia Pathological Society, etc., etc. Philadelphia: Henry C. Lea, 1869. Cloth $7.50, sheep $8.50. Pp. 1,228. Chicago: W. B. Keen & Cooke.

THIS is a superb edition of a work which is now classical and standard. It is unnecessary to say that it is pre-eminent among all works on the subject. Comprehensive as were the previous editions, the present one has been greatly enlarged, and with the judicious and valuable additions of the American editor, constitutes in itself a sufficient encyclopædia of surgical science and art. It has been remodeled and whole chapters rewritten. Many of the old wood-cuts have been withdrawn, and nearly a hundred new ones of greater value introduced. Not the least valuable part of the book is the copious index, which enables the busy practitioner to refer at a glance to the subject which he wishes to examine.

*Third Annual Report of the Metropolitan Board of Health of the State of New York*, 1868. Albany: Printing House of Charles Van Benthuysen & Sons. Pp. 635.

THIS Report is one of the most valuable contributions to sanitary science. It illustrates the liberality and wisdom of the great State of New York in a manner which we should like to see imitated in the great States of the Northwest. In addition to the usual matters of such reports, it contains an exhaustive examination of the "Cattle Disease," "Texas Fever," etc., which has caused such great anxiety during a few years past. The lesions taking place are finely illustrated by a large number of splendid chromo lithographs.

*A Guide Book to Florida and the South*, for Tourists, Invalids and Emigrants. With a Map of the St. John River. By DANIEL G. BRINTON, A.M., M.D. Philadelphia: George Maclean, 719 Sansom Street, 1869. Pp. 136. $1.

FOR those contemplating a sojourn in the South during the cold season, this little book will be found to contain many useful details, as to preparation for the journey, what to take along, routes, hotels, prices, etc., etc.

*Circular No. 2. War Department, Surgeon-General's Office, Washington, January 2, 1869.* A Report on Excisions of the Head of the Femur. for Gun-Shot Injury. Washington: Government Printing Office.

THE Medical Department of the Army, through its most efficient officers, is making contributions to our profession unprecedentedly rich. Circular No. 2 is a report on the subject named in the title, by Assistant-Surgeon George A. Otis, who introduces his work with a full history of the operation.

He then gives, first, a detailed account of the sixty-three excisions of "the head, or of the head, neck, and trochanters," which are all that are sufficiently authenticated, in all particulars, "to make them available for statistical purposes." Then follows a brief notice of the "rejected cases." "Of the sixty-three operations, forty-eight were performed by Union, and fifteen by Confederate surgeons."

The cases are divided into primary, intermediate and secondary operations, a division the importance of which will be recognized by every surgeon of experience. Primary operations were thirty-two; intermediate, twenty-two; and secondary, nine in number.

The primary operations were all made within twenty-four hours of the reception of the injury. Two were successful. Mean duration of life in the thirty unsuccessful cases, was "a little over seven days." One patient struggled out sixty days, which materially increases the average.

The intermediate operations were made between the completion of the second and the twenty-eighth days after the injury. Two of these were successful. Average duration of life in the unsuccessful cases, was twelve and a half days.

Of the secondary operations, one was successful. Mean duration of life in the eight unsuccessful cases was sixteen days, while one poor fellow held out one hundred days.

The interval of time which elapsed between the reception of the injury and the operation averaged two and a half months.

Seventy pages of the report are devoted to a consideration of the subject of "excisions at the hip, compared with temporization," in which the reporter says: "Expectant treatment is to be condemned in all cases in which the diagnosis of direct injury to the articulation can be clearly established."

---

### Pamphlets.

*Advice on the Uses and Abuses of Spectacles and Weak Sight.* By Dr. John Phillipps. Pp. 94. Published by the author, 168 Clark Street, Chicago. Price, twenty-five cents.

A useful monograph on the subject, illustrated by wood-cuts, and containing valuable hints to persons with impaired sight.

*On External Perineal Urethrotomy; or, an Improved Method of External Division of the Urethra in Perineo, for the Relief of Obstinate Stricture.* With remarks on the preparatory and after treatment. By J. W. S. Gouley, M.D., Professor, etc., University of New York, Surgeon to Bellevue Hospital. D. Appleton & Co., New York. From the author.

*The Annals of Iowa.* Published quarterly by the State Historical Society, at Iowa City. July, 1869. Edited by Sanford W. Huff, M.D., Corresponding Secretary.

*Transactions of the Medical Association of the State of Alabama.* Annual Session, 1869, at Mobile, with proceedings of the meeting held for reorganization, at Selma, March, 1868.

*Hygiene in its Relations to Therapeusis.* A paper read before the New York Journal Association by Alfred L. Carroll, M.D.

*Transactions of the Indiana State Medical Society*, at its Nineteenth Annual Session, held at Indianapolis, May, 1869.

*Fœticide, or Criminal Abortion.* A lecture introductory to the course on Obstetrics and Diseases of Women and Children, University of Pennsylvania. Session 1839–40. By Hugh L. Hodge, M.D. Philadelphia: Lindsay & Blakiston, 1869. Chicago: W. B. Keen & Cooke. Paper 30c., cloth 50c.

*Carbolic Acid—Its Action and Uses.* By Charles F. J. Lehlbach, M.D., of Newark, New Jersey. Reprinted from the transactions of the Medical Society of New Jersey, 1869.

*Surgery of the Cervix, in connection with the treatment of certain Uterine Diseases.* By Thomas Addis Emmet, M.D. Read before the Medical Society of the County of New York, February, 1869. From the author.

*Catalogue of the Graduates of Jefferson Medical College, of Philadelphia*, 1826—1869. Ledger Job Printing Office.

*Transactions of the Medical Society of the State of Pennsylvania*, at its Twentieth Annual Session, held at Erie, June, 1869. Fifth series. Part Second. Pp. 567.

*Myxoma, or Hyperplasia of the Villi of the Chorion.* By ALEXANDER SINCLAIR, M.D., etc. Boston: David Clapp & Son, 334 Washington Street. 1869. From the author.

*Puerperal Eclampsia.* By C. C. F. GAY, M.D. Buffalo, August, 1869. From the author.

*The Half-Yearly Abstracts of the Medical Sciences.* Being a Digest of British and Continental Medicines, and of the Progress of Medicine and the Collateral Sciences. Vol. XLIX. July, 1869. Philadelphia: Henry C. Lea, 1869. Pp. 293. $2.50 per annum, in advance. Single copies, $1.50.

*Half-Yearly Compendium of Medical Sciences.* Part IV. July, 1869. Pp. 321. Philadelphia: S. W. Butler, M.D., 115 South Seventeenth Street. $3 per annum, in advance. Single numbers, $2. For 1868 and 1869, Nos. 1 to 4, inclusive, $5.

*The Physician's Visiting List for* 1870. Nineteenth year of its publication. Philadelphia: Lindsay & Blakiston. Chicago: W. B. Keen & Cooke.

